# Role of β‑Arrestin2 Signaling in Neuropharmacological Effects of Psychedelic Drugs

**DOI:** 10.1021/acschemneuro.6c00138

**Published:** 2026-07-27

**Authors:** Aurelija Ippolito, Agnese Tiranti, Shaun Hurley, Gary Gilmour, Grant Churchill, Trevor Sharp

**Affiliations:** † Department of Pharmacology, Mansfield Road, Oxford OX1 3QT, U.K.; ‡ Compass Pathways Plc, London W1F 0DQ, U.K.

**Keywords:** 5-HT_2A_ receptor, psychedelic drugs, β-arrestin2 signaling, neuroplasticity, biased signaling, head-twitch response

## Abstract

Current research into the actions of psychedelic drugs is focused on 5-HT_2A_ receptor-coupled signaling pathways, particularly β-arrestin2 signaling. Here, we investigated the involvement of β-arrestin2 in psychedelic drug-induced mouse markers of hallucinogenic effects, molecular plasticity, and structural plasticity; specifically, the head-twitch response, cortical expression of plasticity-related genes (*cFos*, *Egr1*, *Egr2*, and *Arc*), and dendritogenesis (primary cortical neuron cultures), respectively. Psilocin-induced head-twitches, plasticity gene expression, and dendritogenesis were all blocked by the 5-HT_2A_ receptor antagonist MDL-100,907. The head-twitch response to psilocin was not different between wildtype and β-arrestin2 knockout (KO) mice, and the head-twitch responses to 2,5-dimethoxy-4-iodoamphetamine (DOI) and lysergic acid diethylamide (LSD) were similarly unaffected by β-arrestin2 KO. Psilocin-evoked gene expression showed a trend to be less in β-arrestin2 KO compared to wildtype mice, but the gene expression response to DOI was clearly not altered by β-arrestin2 KO. Finally, psilocin-induced dendritogenesis was attenuated in β-arrestin2 KO versus wild-type cultured neurons, and similar findings were obtained with DOI. In summary, the current study found no convincing evidence to support a role for β-arrestin2 signaling in either the head-twitch or plasticity-related gene responses to the psychedelic drugs tested. However, our data suggest a role for the β-arrestin2 pathway in psychedelic drug-evoked dendritogenesis of cultured neurons. Thus, the β-arrestin2 pathway unlikely mediates the hallucinogenic effects of psychedelic drugs but may contribute to neural plasticity changes in certain models.

## Graphical abstract

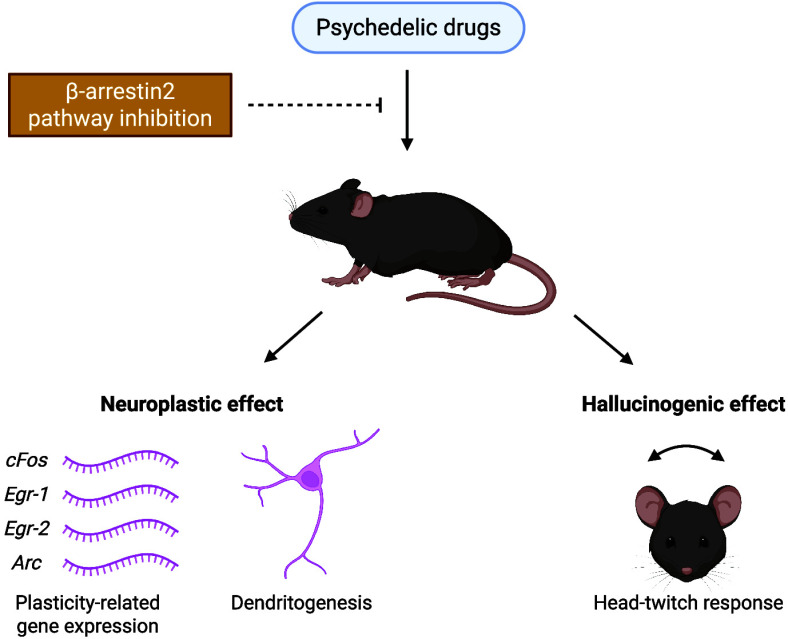

There is much interest in the use of psychedelic drugs to treat psychiatric disorders including major depression and anxiety.^1,2^ Current investigations of the mechanism of psychedelic drugs are focused on the role of the 5-HT_2A_ receptor and its associated signaling pathways.

There is general agreement that the hallucinogenic action of psychedelic drugs is mediated by the 5-HT_2A_ receptor. For example in humans, the subjective effects of psilocybin (active molecule psilocin) and lysergic acid diethylamide (LSD) were blocked by pretreatment with the 5-HT_2_ receptor antagonist, ketanserin.^3,4^ Also, clinical studies using the PET 5-HT_2A_ receptor radioligand [^11^C]­Cimbi-36, report a correlation between 5-HT_2A_ receptor occupancy and the intensity of psilocybin’s subjective effects.^5^ In support of these findings, there is a wealth of evidence from preclinical studies that the head-twitch response to psychedelic drugs, a rodent proxy of the human hallucinogenic effect, is 5-HT_2A_ receptor-mediated.^6^

Much evidence also supports a key role for the 5-HT_2A_ receptor in psychedelic drug-induced neuroplastic effects which are widely considered central to the therapeutic effects of these drugs.^7,8^ For example in preclinical studies, both pharmacological blockade and genetic knockout (KO) of the 5-HT_2A_ receptor were reported to prevent the increase in dendritogenesis evoked by psychedelic drugs, both in primary cultured neurons and *in vivo*.^9–14^ Similar manipulations were also found to prevent psychedelic drug-induced mRNA expression of plasticity-related genes such as *c-Fos*, *Arc* and *Egr1/2*,^15–22^ which each encode activity-dependent transcription factors or proteins involved in various aspects of neuroplasticity such as regulation of synaptic number and strength, spine density and morphology, memory and LTP.^23–33^ There are, however, two studies finding that 5-HT_2A_ receptor antagonists did not block psychedelic drug-induced dendritogenesis,^34,35^ as well as evidence that this effect was mediated by a direct action on TrkB.^35^ On the other hand, the adequacy of antagonist dose in such studies has been questioned,^7^ and a recent study failed to confirm an action of psychedelic drugs on TrkB.^36^

The 5-HT_2A_ receptor couples to both canonical *G*_q_ and noncanonical β-arrestin2 signaling pathways. In the context of the emergence of 5-HT_2A_ receptor agonists with biased signaling properties^37–40^ a key question is, which 5-HT_2A_ receptor-linked signaling pathway(s) mediate hallucinogenic and neuroplastic effects of psychedelic drugs? Support for a role of *G*_q_ signaling in the head-twitch response and neuroplasticity effects of psychedelic drugs comes from several findings. Specifically, it is reported that both the head-twitch and plasticity gene responses to psychedelic drugs were reduced by inhibitors of *G*_q_ and phospholipase C^19,39^ as well as inhibitors of inositol monophosphatase,^41^ which likely reduces *G*_q_ signaling through inositol depletion of the phosphoinositide pathway.

However, in contrast to findings with *G*_q_ signaling, the role of β-arrestin2 in the effects of psychedelic drugs is currently uncertain. In particular, there is conflicting evidence regarding the role of β-arrestin2 in the head-twitch response. Thus, some studies report that β-arrestin2 KO mice had a reduced head-twitch response to LSD and the 5-HT precursor 5-HTP^42,43^ while others found no reduction in the response to 2,5-dimethoxy-4-iodoamphetamine (DOI) and an increased response to 5-MeO–DMT.^42,44^ Moreover, to our knowledge, the role of β-arrestin2 in the neuroplastic effects of psychedelic drugs has not yet been investigated.

Here we used β-arrestin2 KO mice to help fill the gap in knowledge regarding the role of β-arrestin2 signaling in psychedelic drug action. Specifically, we used this mouse to examine the role of β-arrestin2 in psychedelic drug-induced head-twitch responses, cortical expression of plasticity-related genes, and dendritogenesis in primary cortical neuron cultures.

## Results and Discussion

### Psilocin-Induced Head Twitch Response

Initial experiments confirmed that psilocin evoked head-twitches in male and female wildtype (WT) mice. Specifically, psilocin administration (1, 2, or 5 mg/kg i.p.) significantly increased the number of head-twitches compared to vehicle at all doses (*F*_3,56.033_ = 51.634, *p* < 0.0001; Figure 1A), albeit with an inverted U-shaped dose–response as previously observed.^45^ Experiments revealed no consistent difference between male and female mice (for statistical summary see Supporting Information).

**Figure 1:**
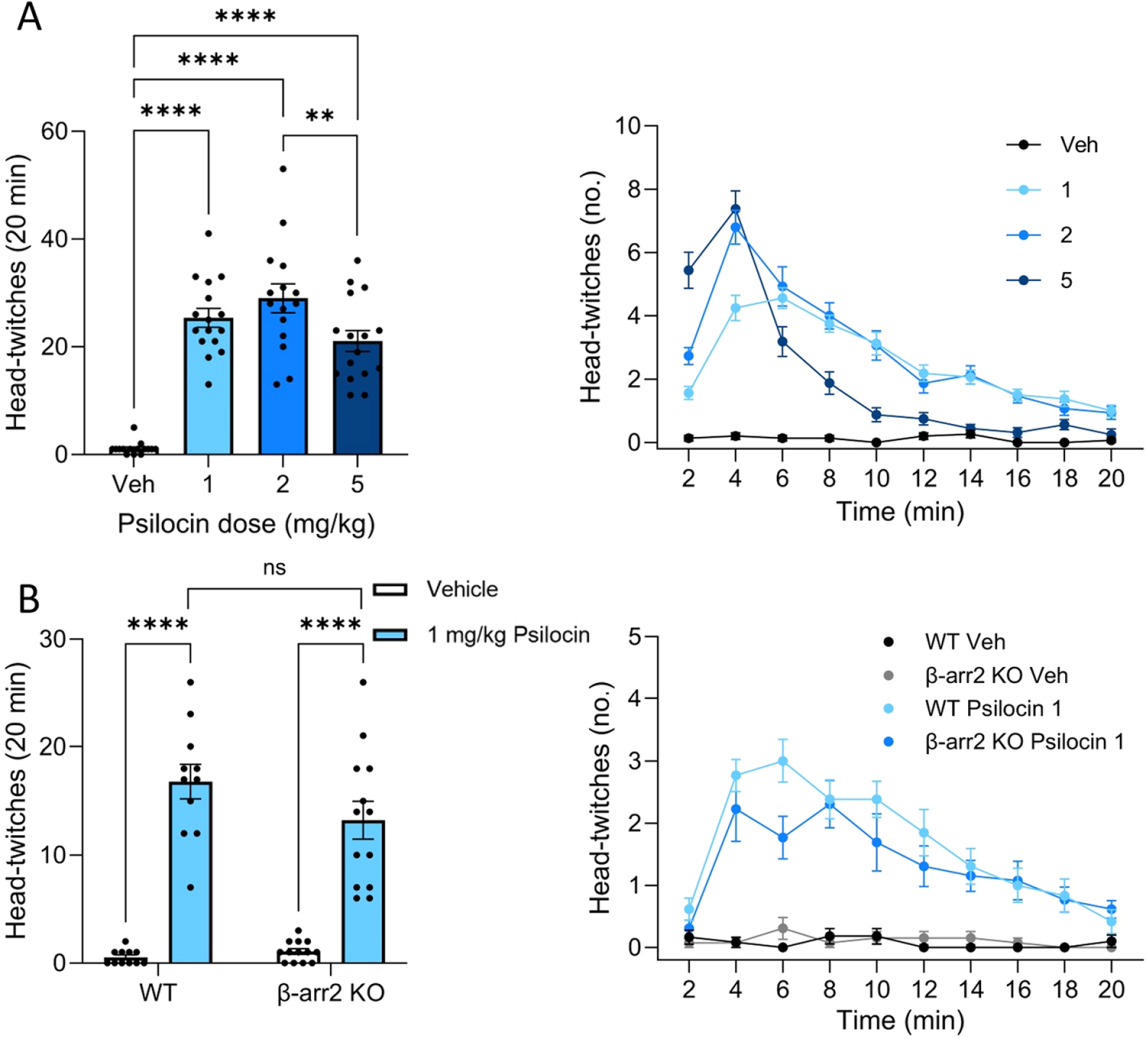
Psilocin-induced head-twitches in WT and β-arrestin2 KO mice. (A) Effect of psilocin (1, 2, or 5 mg/kg i.p.) and vehicle on head-twitches in WT mice (*n* = 14–16). (B) Effect of psilocin (1 mg/kg i.p.) on head-twitches in WT and β-arrestin2 KO mice (*n* = 13–14). Columns show mean ± SEM values with individual data points indicated. Data were analyzed using a mixed effect model and type III ANOVA, with posthoc Tukey’s or Šídák’s tests. **p < 0.01, ****p < 0.0001, ns = not significant.

A separate experiment showed that the head-twitch response to 1 mg/kg i.p. psilocin was abolished by the selective 5-HT_2A_ receptor antagonist, MDL-100,907 (0.3 mg/kg i.p.) (*p* < 0.0001; Supporting Figure 1). This finding agrees with numerous reports that the 5-HT_2A_ receptor mediates the head-twitch response to psilocin and other psychedelic drugs such as DOI and LSD.^19,20,46–50^ Although not tested here, the likely explanation for the U-shaped head-twitch dose–response to psilocin is activity of higher doses of the drug at 5-HT receptors (5-HT_1A_, 5-HT_2C_) which are reported to inhibit 5-HT_2A_ receptor function.^48,51–54^

### Effect of β-Arrestin2 KO on Psilocin-Induced Head Twitches

Next, the role of β-arrestin2 in psilocin-induced head-twitches was investigated. Compared to vehicle, psilocin (1 mg/kg i.p.) increased head-twitches in both wildtype (WT) and β-arrestin2 KO mice (*p* < 0.0001; Figure 1B). Statistical analysis revealed no effect of genotype or a genotype x drug interaction (*F*_1,43.161_ = 1.549, *p* = 0.220 and *F*_1, 43.122_ = 2.958, *p* = 0.093, respectively). Also, there was no significant effect of genotype, genotype x time point or genotype x drug interaction (*F*_1,46.60_ = 1.702, *p* = 0.198; *F*_9,411.05_ = 0.680, *p* = 0.727; and *F*_1, 46.55_ = 2.340, *p* = 0.133, respectively) (Figure 1B).

Given the finding in a previous study that β-arrestin2 KO mice had a reduced head-twitch response to LSD,^43^ we next validated the KO mice by showing that β-arrestin2 expression was undetectable (Figure 2A) and without compensatory upregulation of β-arrestin1 expression (Figure 2B). There was also no change in 5-HT_2A_ receptor expression in β-arrestin2 KO versus WT mice (Figure 2C). It is possible that surface expression or functional coupling of the 5-HT_2A_ receptor may be altered in β-arrestin2 KO mice. Additionally, given that other 5-HT receptors can modulate 5-HT_2A_ receptor signaling and functional effects, it is also possible for the loss of β-arrestin2 to impact on those modulatory processes. However, similar results to psilocin in subsequent head-twitch experiments using DOI and LSD (see below) likely rule out these potential confounds.

**Figure 2:**
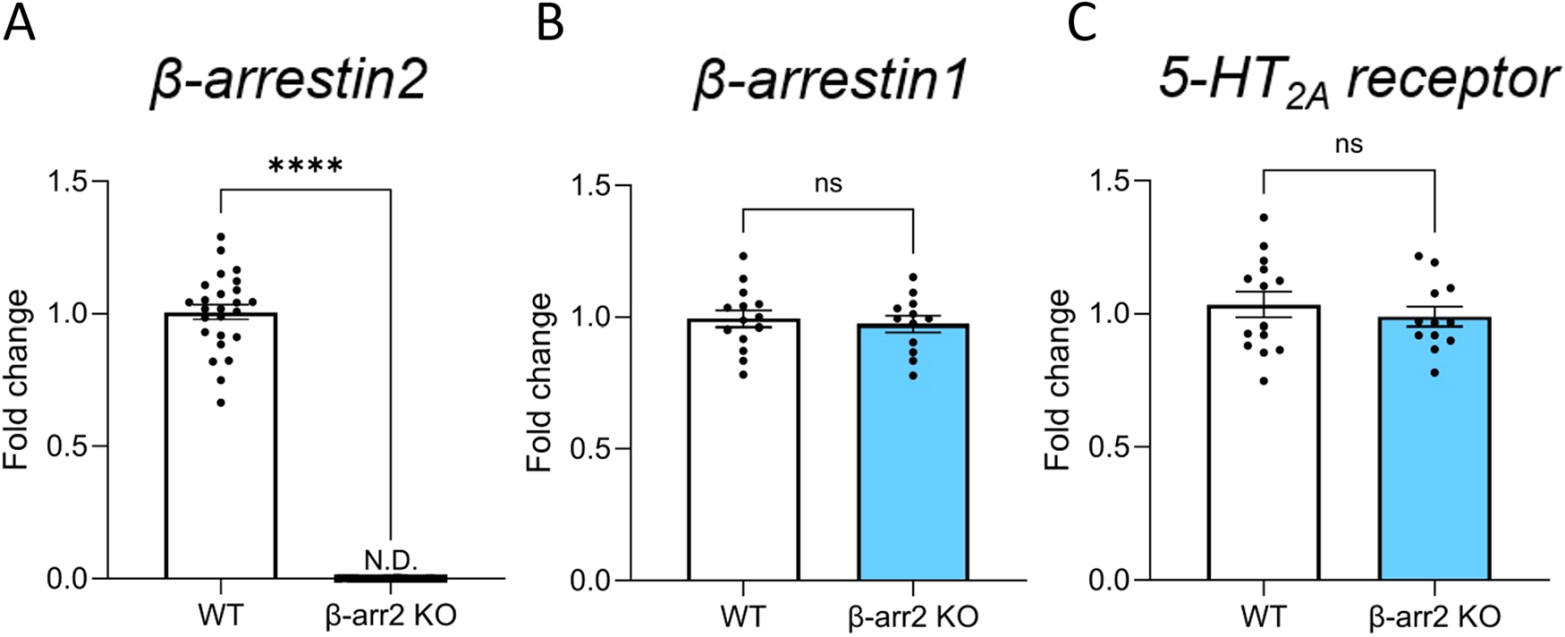
Molecular validation of β-arrestin2 KO mice versus WT controls. mRNA encoding (A) β-arrestin2 (*n* = 25–33), (B) β-arrestin1 (*n* = 12–14) and (C) 5-HT_2A_ receptor (*n* = 12–14) in WT and β-arrestin2 KO mice. Columns represent mean ± SEM values, with individual data points indicated. Data were analyzed using an unpaired Student’s *t* test. ****p < 0.0001, ns = not significant, N.D.=not detectable.

### Effect of β-Arrestin2 KO on DOI- and LSD-Induced Head Twitches

Involvement of β-arrestin2 in DOI-induced head-twitches was explored as a comparison to psilocin. Administration of 2 mg/kg DOI increased head-twitches in both WT and in β-arrestin2 KO mice (*p* < 0.0001; Figure 3A). There was no effect of genotype on number of head-twitches nor a drug x genotype interaction (*F*_1,29.515_ = 0.371, *p* = 0.547 and *F*_1, 29.540_ = 0.0450, *p* = 0.833, respectively; Figure 3A). Over the time-course there was also no effect of genotype nor a significant genotype *x* time or genotype x drug interaction (*F*_1,319.980_ = 0.00280, *p* = 0.958 and *F*_1,319.140_ = 0.0018, *p* = 0.9660, respectively; Figure 3A). In agreement, it was previously reported that the head-twitch response to 1 mg/kg DOI was not different between β-arrestin2 KO and WT mice.^44^

**Figure 3:**
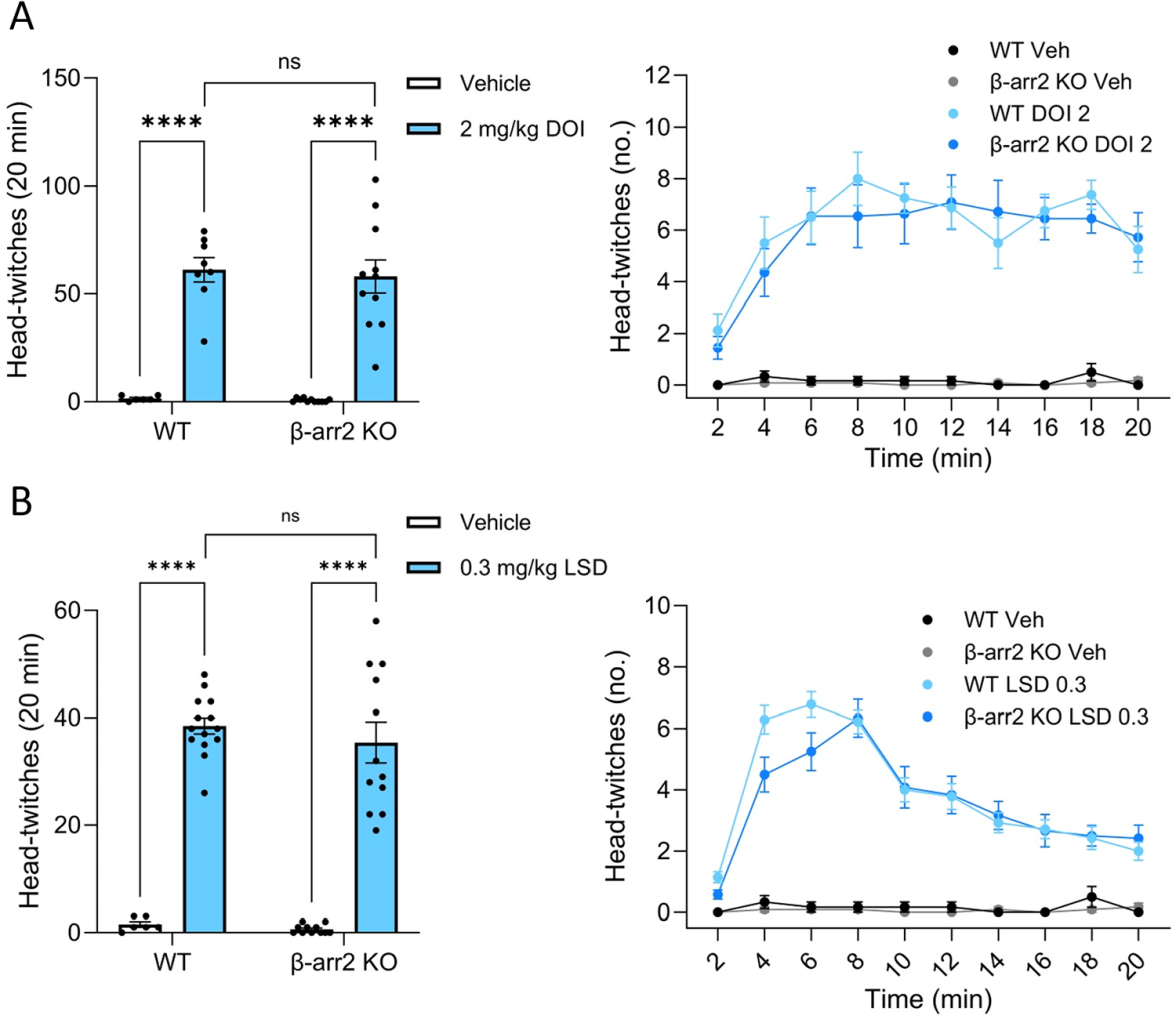
Psychedelic drug-induced head-twitches in WT and β-arrestin2 KO mice. Effect of 2 mg/kg DOI versus vehicle (A) and 0.3 mg/kg LSD versus vehicle (B) on head-twitches in WT and β-arrestin2 KO mice compared to vehicle (left, head-twitches accumulated over 20 min; right, time-course over 20 min). Columns represent mean ± SEM values, with individual values indicated by symbols (vehicle *n* = 6–11, DOI *n* = 8–11, LSD *n* = 12–14). Data were analyzed by a mixed effect model and type III ANOVA and if significant, followed by posthoc Tukey’s Honest Significant Difference tests. ****p < 0.0001, ns = not significant.

Given a previous report of a reduced head-twitch response to LSD in β-arrestin2 KO mice,^43^ LSD was also tested here. Both WT and β-arrestin2 KO mice displayed head-twitches in response to 0.3 mg/kg LSD (*p* < 0.0001; Figure 3B) but there was no significant effect of genotype nor a drug x genotype interaction (*F*_1,36.274_ = 0.6249, *p* = 0.4344 and *F*_1,37.077_ = 0.0972, *p* = 0.7570, respectively; Figure 3B). Over the time-course there was also no effect of genotype nor genotype *x* time or genotype *x* drug interactions (*F*_1,387.84_ = 1.4845, *p* = 0.224, *F*_9,386.95_ = 1.1404, *p* = 0.333 and *F*_1,388.16_ = 0.2524, *p* = 0.616, respectively; Figure 3B). Therefore, our results were unable to confirm that β-arrestin2 KO reduced the head-twitch response to LSD as previously reported.^43^

It is notable that the mean number of head-twitches in response to LSD in WT mice here was approximately double of that reported in WT mice in the previous study.^43^ Although the strain of WT and β-arrestin2 KO mice used here and previously were the same, it is theoretically possible that baseline differences in the head-twitch response to LSD may have prevented detection of an inhibitory effect of β-arrestin2 KO. However, our results with LSD are in accord with the lack of effect of β-arrestin2 KO on the head-twitch responses to both psilocin and DOI.

Thus, data here provides no support for a role of β-arrestin2 in the head-twitch response to psilocin, DOI or LSD. Alternatively, *G*_q_ signaling has been implicated in psychedelic drug-evoked head-twitches. Thus, the potency of 5-HT_2A_ receptor agonists to induce head-twitches was correlated with *G*_q_ but not β-arrestin2 signaling efficacy *in vitro*.^39^ Moreover, DOI-induced head-twitches were inhibited by pretreatment with the *G*_q/11_ inhibitor, YM-254890 or with the phosphoinositide-selective PLC inhibitor edelfosine.^39^ Additionally, *G*_q_ KO mice showed reduced head-twitches to DOI, and both DOI- and psilocin-induced head-twitches were reduced by the inositol-monophosphatase inhibitors, ebselen and lithium (which decrease *G*_q_ signaling).^41,55^ Collectively, these findings constitute strong support for a key role of *G*_q_ signaling in 5-HT_2A_ receptor-induced head-twitches. Although the present data exclude involvement in the head-twitch response of β-arrestin2, other signaling pathways have been linked to the 5-HT_2A_ receptor including phospholipase A, and further signaling pathways potentially contributing the head-twitch response may be discovered.

### Effect of Psilocin on Plasticity-Related Gene Expression

There is general agreement that increased neural plasticity plays a central role in the therapeutic effects of psychedelic drugs. However, the 5-HT_2A_ receptor-linked signaling pathways mediating plasticity changes are yet to be clearly defined. Here, administration of psilocin (1, 2, or 5 mg/kg i.p.) increased mRNA expression of a panel of plasticity-related genes (*c-Fos*, *Egr-1*, *Egr-2*, *Arc*) in frontal cortex of WT mice, with the effect greatest at the 1 mg/kg dose (*c-Fos* F_3, 55.009_ = 4.759, *p* = 0.00508; *Egr-1* F_3, 53.039_ = 3.607, *p* = 0.0191; *Egr-2* F_3, 53.057_ = 5.266, *p* = 0.00297; *Arc* F_3, 56.000_ = 3.9033, *p* = 0.0133; Figure 4A, and Supporting Table 1). Pretreatment with 0.3 mg/kg MDL-100,907 blocked the effect of 1 mg/kg psilocin (*c-Fos* *p* = 0.0020, *Egr-1* *p* = 0.0002, *Egr-2* *p* = 0.0004, *Arc* *p* < 0.0001; Figure 4B) confirming 5-HT_2A_ receptor involvement. These results are consistent with previous studies reporting that psilocin, DOI and LSD increased expression of *c-Fos*, *Egr-1* and *Egr-2* and *Arc* in various mouse brain regions^19,41,56^ and that such effects were abolished by 5-HT_2A_ receptor blockade or KO.^19,20,56^

**Figure 4:**
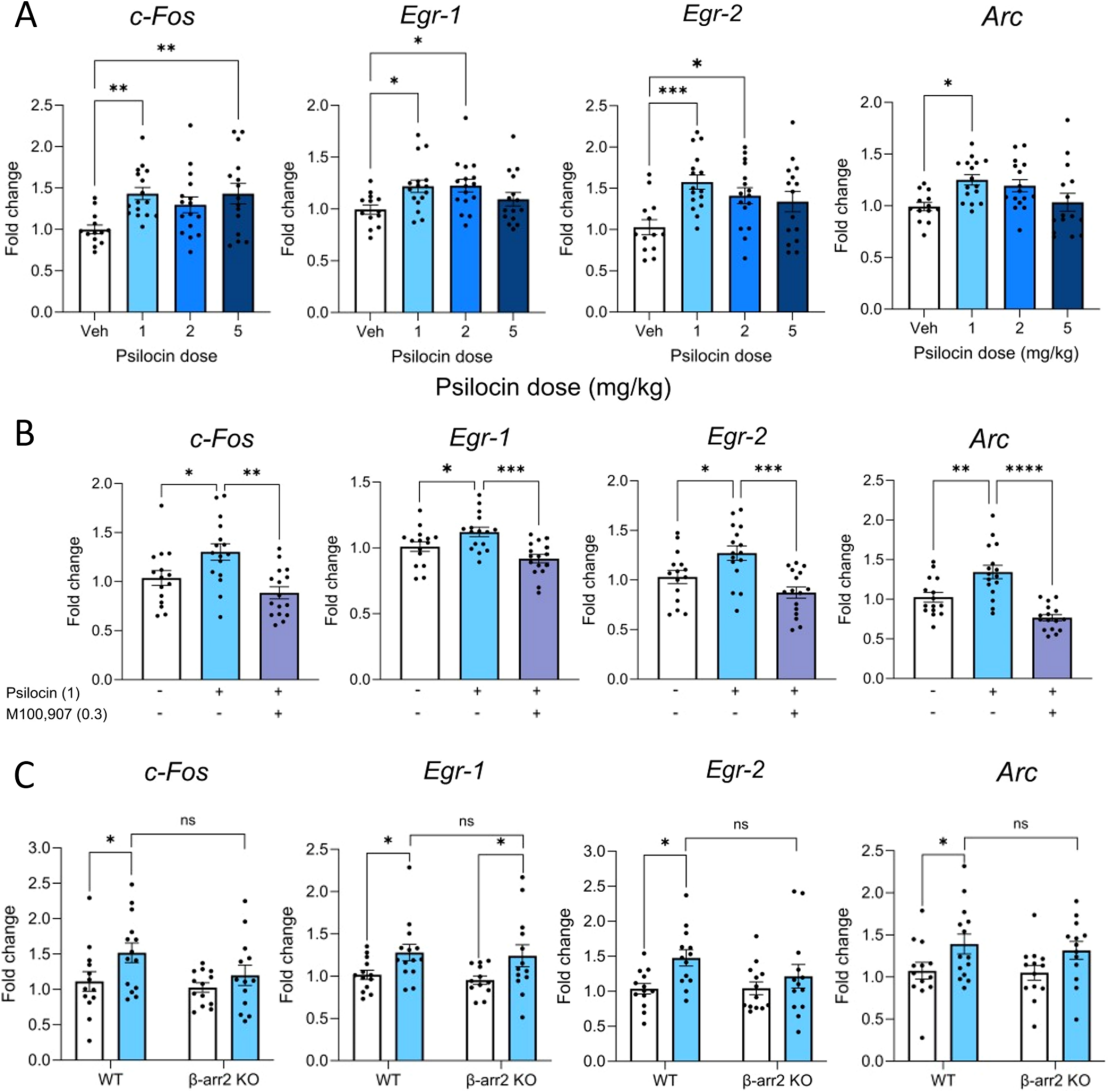
Effect of psilocin on mRNA expression neural plasticity genes in mouse frontal cortex. Effect of (A) dose response to psilocin (1, 2, 5 mg/kg) versus vehicle in WT mice (*n* = 14–16), (B) psilocin (1 mg/kg) after pretreatment with MDL-100,907 (0.3 mg/kg) in WT mice (*n* = 15–16), (C) vehicle (open column) versus psilocin (1 mg/kg; blue column) in WT and β-arrestin2 KO mice (*n* = 13–14). Columns represent mean ± SEM values, with individual values indicated by symbols. Data were analyzed by a mixed effect model and type III ANOVA and if significant, followed by posthoc Tukey’s Honest Significant Difference or Šídák’s tests. *p < 0.05, **p < 0.01, ***p < 0.001, ****p < 0.0001, ns = not significant.

Here, mRNA expression was measured 60 min postinjection, a time point that likely captures acute transcriptional responses to psychedelic drug administration rather than sustained plasticity-related gene regulation. However, as mentioned in the Introduction, each of *Fos*, *Egr-1*/2 and *Arc* are associated with promoted structural and functional plasticity changes, thus their mRNA upregulation likely provides an indirect readout of eventually sustained neuroplasticity.

### Effect of β-Arrestin2 KO on Psilocin-Evoked Increase in Plasticity-Related Gene Expression

Next, the role of β-arrestin2 in the plasticity gene response to psilocin was investigated. As observed above, compared to vehicle controls 1 mg/kg psilocin increased plasticity gene expression in WT mice (*c-Fos*, *p* = 0.0275; *Egr-1*, *p* = 0.00390; *Egr-2*, *p* = 0.0123; *Arc*, *p* = 0.00599; Figure 4C). Importantly, this effect of psilocin was not significantly different in β-arrestin2 KO mice in that there was no effect of genotype or genotype *x* drug interaction in any of *c-Fos* (effect of genotype: *F*_1,49.000_ = 2.541, *p* = 0.117; interaction: *F*_1,49.000_ = 0.840, *p* = 0.364), *Egr-1* (F_1,48.000_ = 0.314, *p* = 0.578; *F*_1,48.000_ = 0.0169, *p* = 0.897), *Egr-2* (*F*_1,47.000_ = 1.169, *p* = 0.285; *F*_1,47.000_ = 1.321, *p* = 0.256) or *Arc* (*F*_1,48.814_ = 0.350, *p* = 0.557; *F*_1, 46.227_ = 0.0704, *p* = 0.792). However, visual inspection of the data suggested that for some genes (*c-Fos, Egr-2*) the effect of psilocin was smaller in β-arrestin2 KO versus WT mice (Figure 4C, and Supporting Table 2). Calculations showed that the group sizes used (*n* = 13–14) were sufficiently powered to detect a reduction in gene expression (including *cFos* and *Egr-2*) of ∼30% in the β-arrestin2 KO mice, suggesting that the findings were reliable.

### Effect of β-Arrestin2 KO on DOI-Evoked Increase in Plasticity-Related Gene Expression

Given the trend for a reduced effect psilocin on gene expression in β-arrestin2 KO mice, we also tested the plasticity gene response to DOI which our previous studies have shown to increase plasticity gene expression through a 5-HT_2A_ receptor-mediated mechanism.^20^ In WT mice, administration of DOI (2 mg/kg) increased mRNA expression of each gene tested (*p* < 0.0001 versus vehicle in all cases). Furthermore, as observed with psilocin, the effect of DOI on gene expression was not significantly different between WT and β-arrestin2 KO mice (Figure 5, and Supporting Table 3). Thus, there was no effect of genotype or genotype x drug interaction for *c-Fos* (effect of genotype: *F*_1,32.000_ = 0.403, p = 0.530; interaction: *F*_1,49.000_ = 0.840, *p* = 0.364), *Egr-1* (F_1,31.997_ = 2.256, *p* = 0.143; F_1,31.678_ = 0.930, *p* = 0.342), *Egr-2* (*F*_1, 32.000_ = 2.062, *p* = 0.161; *F*_1,32.000_ = 0.655, *p* = 0.424) or *Arc* (F_1, 32.000_ = 0.297, *p* = 0.590; *F*_1, 46.227_ = 0.0193, *p* = 0.890).

**Figure 5:**
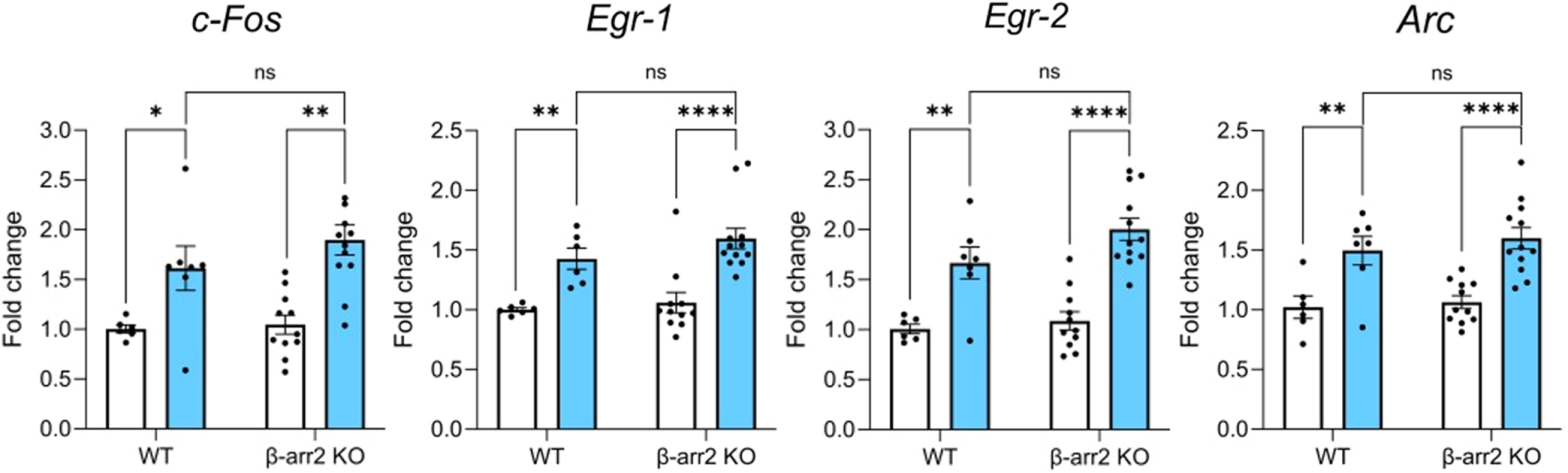
Effect of DOI on mRNA expression neural plasticity genes in mouse frontal cortex. DOI (2 mg/kg) or vehicle (*n* = 6–11 and *n* = 8–11, respectively) were administered to WT and β-arrestin2 KO mice. Columns (open = vehicle, blue = DOI) represent mean ± SEM values, with individual values indicated by symbols. Data were analyzed by a mixed effect model and type III ANOVA and if significant, followed by posthoc Šídák’s tests. *p < 0.05, **p < 0.01, ****p < 0.0001, ns = not significant.

Taking the psilocin and DOI data together, we conclude from these experiments that β-arrestin2 signaling does not play an important role in psychedelic drug-induced plasticity gene expression, and that *G*_q_ signaling is more likely to play a role. In keeping with the latter, it was reported previously that DOI-evoked plasticity gene expression in mouse cortex was reduced by administration of inositol monophosphatase inhibitors^41^ as well as *G*_q_ KO.^55^ Also, the PLCβ inhibitor U73122 abolished LSD-induced plasticity gene expression changes in mouse/rat primary cortical neurons.^19^

### Effect of Psilocin and DOI on Dendritogenesis in Cultured Cortical Neurons

Finally, experiments investigated the role of β-arrestin2 in psychedelic drug-induced structural plasticity, specifically dendritogenesis in mouse cortical neurons generated using primary cultures. Treatment of cultured neurons for 60 min with either psilocin (10 μM) or DOI (10 μM) had an effect on the maximum number of neurite crossings (Nmax) (*F*_2,270_ = 4.981, *p* = 0.0075), with both psilocin and DOI increasing Nmax compared to vehicle-treated controls (*p* = 0.0026 and *p* < 0.0001, respectively) (Figure 6A). This effect was also evident in the area under the curve (AUC) of the Sholl plot (*F*_2,11217_ = 6.676, *p* = 0.0013), which was increased by both psilocin and DOI (*p* > 0.0001 for both) (Figure 6B). Psilocin and DOI also increased secondary measures of dendritic growth, specifically total number of branches and neurite length (Supporting Figure 2A,B). These findings are in agreement with previous reports of psilocin- and DOI-induced dendritogenesis, both *in vitro* and *in vivo*.^11,12,34,35^

**Figure 6:**
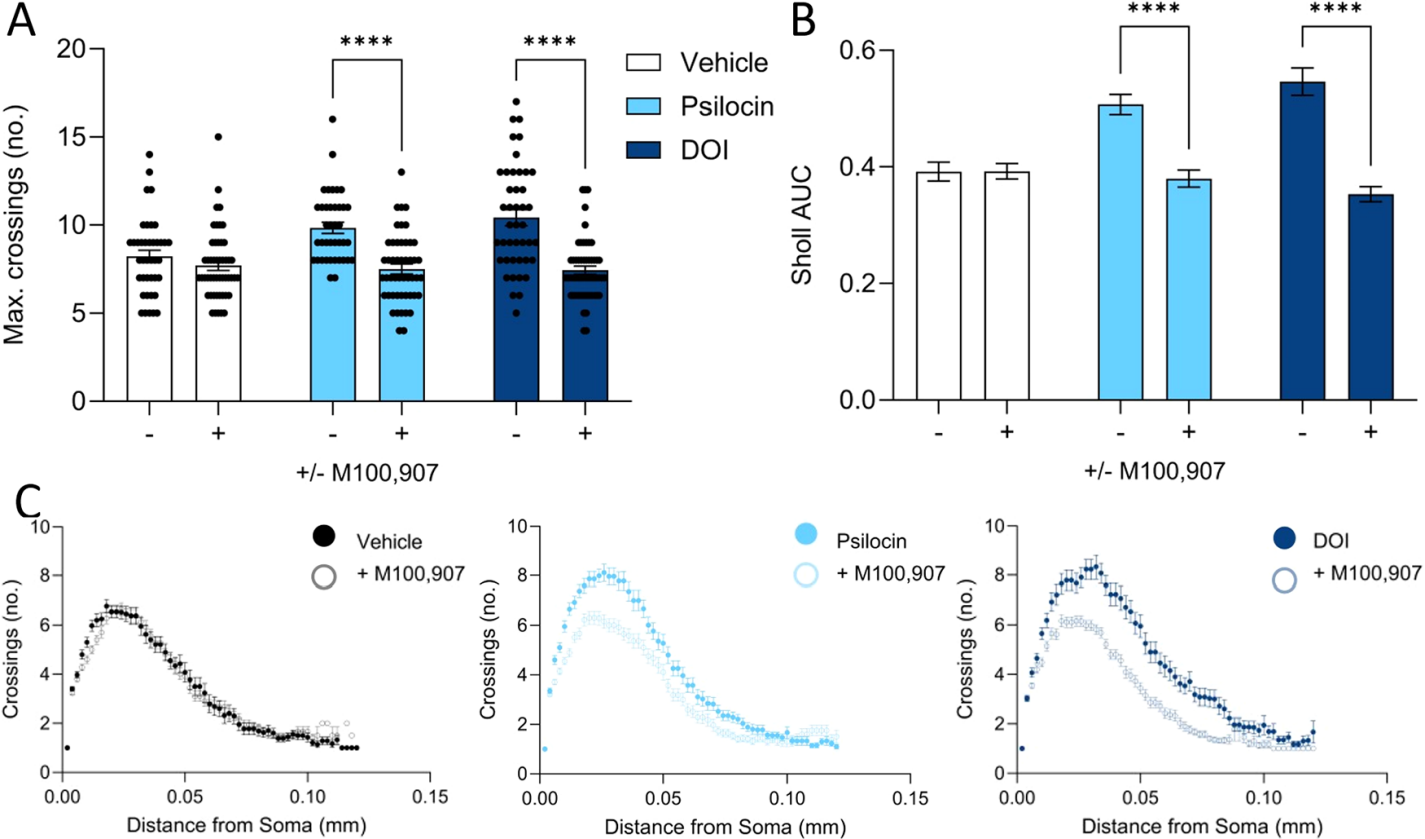
Effect of psilocin and DOI on dendritogenesis of primary cortical neurons of WT mice. (A) Maximum number of crossings and (B) Sholl plot AUC of neurons treated with vehicle, psilocin (10 μM) or DOI (10 μM) in the presence/absence of MDL-100,907 (1 μM). (C) Sholl plots showing number of crossings with increasing distance from soma for vehicle (left), psilocin (middle) and DOI (right) with and without MDL-100,907. Each point is a mean ± SEM value.

The role of the 5-HT_2A_ receptor in the dendritogenesis effect of psilocin and DOI was confirmed using MDL-100,907. Thus, pretreatment (15 min) with MDL-100,907 (1 μM) blocked both psilocin- and DOI-induced dendritogenesis as evident from measures of Nmax (*p* < 0.0001 for both: Figure 6A), Sholl plot AUC (*p* < 0.0001 for both; Figure 6B) as well as number of branches and neurite length (Figure 6B, and Supporting Figure 2A,B).

These data are in accord with earlier evidence that the 5-HT_2A_ receptor antagonist ketanserin or 5-HT_2A_ receptor KO blocked dendritogenesis induced by various psychedelic drugs including LSD and DOI.^11,14^ While a previous study reported that LSD-evoked dendritogenesis was mediated by a direct action on the TrkB receptor,^35^ a recent study failed to detect an interaction between LSD and TrkB.^36^ Taking the current and previous findings together, the balance of evidence is strongly in favor of psychedelic drug-induced dendritogenesis being mediated by the 5-HT_2A_ receptor, at least under the present experimental conditions.

### Effect of β-Arrestin2 KO on Psilocin- and DOI-Induced Dendritogenesis

Experiments next investigated the role of β-arrestin2 in psilocin- and DOI-evoked dendritogenesis. Primary cortical neurons were dissected from WT or β-arrestin2 KO mice and treated with psilocin (10 μM), DOI (10 μM) or vehicle for 60 min. Statistical analysis revealed an effect of drug on Nmax (*F*_2,242_ = 9.749, *p* < 0.0001) and strong trend effects of genotype and genotype x drug interaction (*F*_1,242_ = 3.874, *p* = 0.0502 and *F*_2,242_ = 2.982, *p* = 0.0526, respectively). Posthoc analysis revealed that the Nmax response to psilocin was significantly less in β-arrestin2 KO versus WT neurons (*p* = 0.0479), although this was not the case for DOI (*p* = 0.747) (Figure 7A,C). However, compared to vehicle neither psilocin nor DOI increased Nmax in β-arrestin2 KO neurons (*p* > 0.999 and *p* = 0.233, respectively) (Figure 7A,C).

**Figure 7:**
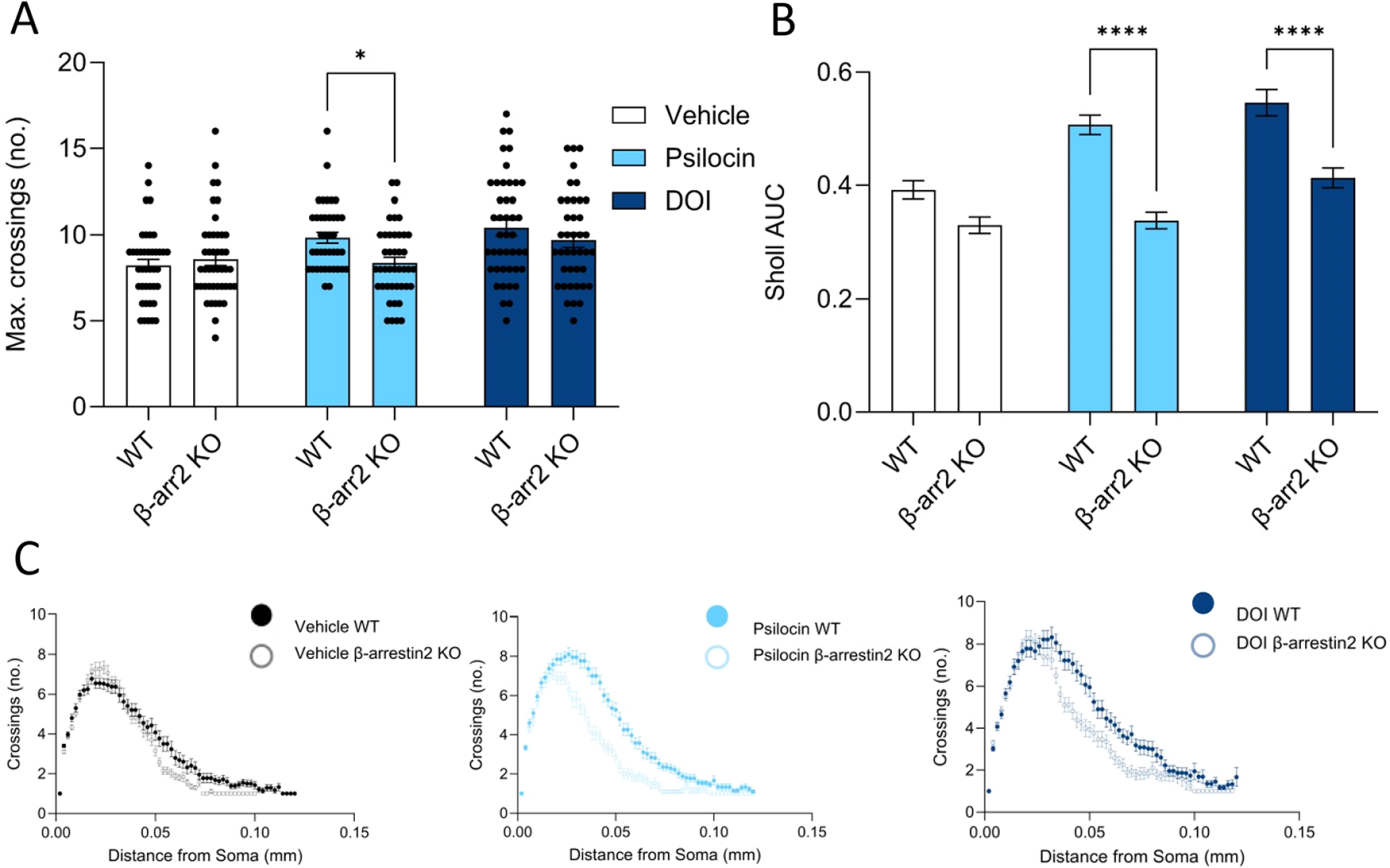
Effect of psilocin and DOI on dendritogenesis of primary cortical neurons of WT and β-arrestin2 KO mice. (A) Maximum number of crossings (B) Area under Sholl plot. *n* = 39–55 neurons. (C) Sholl plots showing number of crossings with increasing distance from the soma for WT and β-arrestin2 KO neurons treated with vehicle (left), psilocin (middle), DOI (right). Each point is the mean ± SD value. Columns represent mean ± SEM values, with individual values indicated by symbols. WT neuron data is the same as in Figure 6. Data were analyzed by a two-way ANOVA and if significant, followed by posthoc Šídák’s multiple comparisons tests. *p < 0.05, **p < 0.01, ***p < 0.001, ****p < 0.0001.

Sholl plot AUC analysis also revealed statistically significant effects of drug, genotype and genotype x drug interaction (*F*_2,9334_ = 19.42, *p* < 0.0001; *F*_1,9334_, *p* < 0.0001 and *F*_29334_ = 4.009, *p* = 0.0182, respectively). Posthoc analysis revealed that the response to both psilocin and DOI was less in β-arrestin2 KO versus WT neurons (*p* < 0.0001 in each case). Compared to vehicle, psilocin did not significantly increase Sholl plot AUC in β-arrestin2 KO neurons and the response to DOI was markedly reduced.

As with the Nmax and Sholl plot AUC data, psilocin- and DOI-induced increases in total number of branches and total neurite length were also significantly reduced by β-arrestin2 KO (Supporting Figure 3A,B).

Overall, the current data show that β-arrestin2 KO reduced psilocin- and DOI-induced measures of dendritogenesis in cultured primary cortical neurons. Although there are no previous studies on the role of β-arrestin2 in the structural plasticity effects of psychedelic drugs, this finding accords with previous *in vivo* work showing that increased measures of structural plasticity in mouse hippocampus induced by the selective 5-HT reuptake inhibitor fluoxetine were attenuated by β-arrestin2 KO.^57^ The downstream signaling pathways by which 5-HT_2A_ receptor-mediated recruitment of β-arrestin2 might induce structural plasticity changes are unknown. However, β-arrestin-dependent activation of mTOR has also been described in neurons,^58–60^ and that pharmacological inhibition of mTOR attenuated dendritogenesis induced by DOI and LSD.^11,61^

It is interesting that the current data suggests a role for β-arrestin2 in psychedelic drug-induced structural plasticity but not expression of plasticity-related genes. One factor that complicates interpretation of these data is that our measure of structural plasticity was obtained from *in vitro* studies of developing cortical neurons whereas the gene expression measurements were obtained from adult mouse cortex *in vivo*, and thus conclusions drawn are model-limited. Although embryonic primary cortical neurons are commonly used to assess psychedelic drug-evoked structural plasticity changes,^11,34,35^ these measures are representative of a different time window of development than is relevant to the therapeutic effects of psychedelic drugs in a clinical context. Future studies measuring both molecular and structural plasticity readouts within the same model will be informative to assess whether there is true mechanistic dissociation between immediate early gene markers and structural plasticity. In support of the latter idea, a recent study reported evidence that even measurements of dendritogenesis and plasticity gene effects of 5-HT_2A_ receptor agonists in adult mice *in vivo* are mechanistically separable.^14^ This adds further to the idea that the plasticity gene and structural plasticity effects of psychedelic drugs involve distinct signaling pathways and should not be considered as equivalent forms of plasticity. Future studies are required to assess the role of β-arrestin2 in antidepressant-like behavioral effects of psychedelic drugs.

## Conclusions

The present study reports evidence that 5-HT_2A_ receptor signaling via β-arrestin2 does not play a role in psychedelic drug-induced head-twitches, and therefore this pathway is not likely to mediate the hallucinogenic effects of these drugs. In comparison, our data suggest that 5-HT_2A_ receptor signaling via β-arrestin2 may contribute to structural plasticity changes but not the expression of plasticity-related genes.

## Methods

### Animals

All mice were group housed (4–6 per cage, 21 °C, 12 h light-dark cycle) in individually ventilated cages lined with sawdust bedding and cage enrichment with ad libitum access to food and water. WT mice (C57BL/6J, 8–9 weeks; Charles River) were used to establish the effects of different doses of psilocin on head-twitches and gene expression. β-arrestin2 KO mice (C57BL/6J background strain with deletion of the *Arrb2* gene through homologous recombination; JAX laboratories) were maintained by Het × Het breeding, and experimental cohorts comprised male and female WT and KO littermates. Experiments followed the principles of the Animal Research: Reporting of In Vivo Experiments (ARRIVE 2.0) guidelines and were conducted according to the United Kingdom Animals (Scientific Procedures) Act of 1986, under project license PP1503281 at the University of Oxford.

### Drugs and Reagents

Solutions for *in vivo* experiments were prepared on the morning of experiment. Psilocin (supplied by Compass Pathways) and MDL-100,907 (volinanserin; Biotechne) were dissolved in 50 mM tartaric acid, then pH was increased to 5.5 using 50 mM tartaric acid and 1 M sodium hydroxide before diluting further in 5% glucose-saline and 0.9% saline to concentrations of 0.1, 0.2, and 0.5 mg/mL (psilocin) and 0.03 mg/mL (MDL-100,907). DOI hydrochloride (Cambridge Bioscience) and LSD (Chiron) were dissolved in 0.9% saline to concentrations of 0.2 and 0.03 mg/mL, respectively. Vehicle consisted of either 50 mM tartaric acid, 1 M sodium hydroxide, 5% glucose-saline and 0.9% saline at pH 5.5 (psilocin and MDL-100,907 experiments) or 0.9% saline (DOI and LSD experiments). All drugs were injected i.p. at a volume of 10 mL/kg.

For experiments on cultured primary neurons, psilocin, DOI and MDL-100,907 were dissolved in DMSO to generate a stock 10 mM solution. On the day of experiment, stock solutions were diluted to working concentrations of 10 μM psilocin and DOI or 1 μM MDL-100,907. Vehicle was 0.1% DMSO.

### Head-Twitch Measurements

On the day of experiment, mice were habituated to the experimental room and their individual cages for 90 min before the first injection. For antagonist experiments MDL-100,907 or vehicle was given 30 min prior to injection of psilocin. Immediately after injection, mice were placed individually in clear cages and recorded by video for 20 min. Head-twitch counts were made by direct observation postexperiment by an experimenter blind to treatment and genotype.

### qPCR Measurement of Gene Expression

Sixty min postinjection mice were cervically dislocated, and whole brain was removed, snap frozen in isopentane, and stored at −80 °C until use. Frontal cortex was dissected, and RNA extraction performed using the TRIzol method^62^ (Qiagen RNeasy Mini Kit, Qiagen) followed by cDNA conversion and subsequent qPCR as described previously.^63^ For each sample, 800 ng of RNA was converted to cDNA using a high-capacity cDNA Reverse Transcription Kit (Life Technologies) and T100 Thermocycler (Bio-Rad). Details of the primer sequences used for qPCR analysis can be found in Supporting Table 4.

For qPCR, a 384-well plate was used with 10 μL reaction volume per well, 20 ng cDNA, 6 pmol forward and reverse primers, 1.3 μL nuclease-free water and 5 μL PrecisionPLUS qPCR Master Mix with SYBRgreen (Primer Design). The qPCR protocol consisted of enzyme activation at 95 °C for 2 min, 40 cycles of 95 °C for 10 s and 60 °C for 1 min, then holding at 4 °C. Each sample was run in duplicate.

qPCR data was analyzed using the Δ*C*_t_ method.^64,65^ Outliers were identified using ROUT analysis (GraphPad Prism) and these samples were excluded from further analysis.

### Mouse Primary Cortical Neuron Cultures

Embryos (E16-E18) were dissected from pregnant WT (C57BL6/J) or β-arrestin2 KO mice following a previous protocol.^66^ Briefly, cortical tissue from embryos were dissected in dissection solution (DS), comprising neurobasal medium, 100 IU μg^–1^ ml^–1^ penicillin/streptomycin, 5 mM Glutamax (GibcoTM) and amphotericin B. Cortices were digested with a solution of 50 U of Papain for 30 min at 37 °C, and digestion stopped by addition of trypsin inhibitor for 10 min. Cells were placed in DS + 2% B27, triturated using a flame-sterilized glass pipet, and filtered via a cell strainer. Cells were then plated at a density of 3 × 10^5^ cells/well in a poly l-lysine coated 6-well plate in 2 mL DS + 2% B27. Cultures were maintained at 37 °C and 5% CO_2_ with half media changes every 2–3 days.

### Dendritogenesis Experiments

Dendritogenesis experiments followed a previously described protocol.^61^ On the third day after primary neuron dissection (DIV3), MDL-100,907 or vehicle were incubated with the cells for 15 min at 37 °C. Next, vehicle, psilocin or DOI were added to cells and incubated for 1 h at 37 °C. After 1 h, media was removed and a mixture of fresh and conditioned media added back to wells. The cells were then allowed to grow for a further 72 h.

On DIV6, media was aspirated from wells and cells rinsed twice with dPBS-Ca^2+^. Cells were then fixed with 4% paraformaldehyde for 20 min, and rinsed three times with dPBS-Ca^2+^. Cells were then permeabilised with 0.3% Triton-X-100 for 5 min at RT and rinsed three times with dPBS-Ca^2+^. Cells were next blocked with 5% goat serum diluted in dPBS-Ca^2+^ (blocking buffer) for 1 h at RT. The blocking buffer was removed from the cells and the primary antibody (rabbit anti-MAP2), diluted 1:1000 in blocking buffer, was added. The coated cells were incubated overnight at 4 °C. Next day, cells were rinsed three times with dPBS-Ca^2+^ before treatment with the secondary antibody (Alexa Fluor 488 goat antirabbit), diluted 1:500 in blocking buffer. Coated cells were incubated for 1 h at RT. Finally, cells were rinsed three times with dPBS-Ca^2+^.

Cells were imaged using a fluorescent microscope (Invitrogen Evos FL Auto 2) with 20× magnification, and imaging settings were kept constant throughout. Image capture was automated, such that 1% of the well area was randomly sampled and imaged. From these images, the neurons analyzed were chosen as those that had the least overlap with neighboring neurons. Neuron selection and the subsequent data analysis was done blind to treatment.

Images were processed using ImageJ^67^ according to a previous protocol.^68^ Images were first converted to 8-bit and grayscale, cleaned, and then converted to binary. From here, any surrounding neurons in the image were erased before the image was skeletonised. The skeletonised image was marked with a circle to indicate the center of the soma. Then, the image was opened in the SNT ImageJ plugin and subjected to autotracing.

Measures of dendritogenesis were obtained by Sholl analysis^69^ (batch analysis script of ImageJ) using a step size radius of 2 μm. From the Sholl plots, measures of the maximum number of crossings (Nmax) of each individual neuron were taken. Additionally, the Sholl plots of all neurons in one treatment group were averaged and the AUC of this averaged plot was determined. Other measures such as total number of branches and total neurite length were also determined.

### Statistical Analysis

GraphPad Prism software (Version 10.4.1) was used for graph plotting and testing of normality with Shapiro-Wilk or D’Agostino and Pearson tests. For head-twitch and gene expression data, RStudio (RStudio 2024.04.1) was used for linear mixed-effects modeling, with sex and experimental cohort treated as covariates, as previously described.^70^ This was followed by Type III Analysis of Variance (ANOVA) Table with Satterthwaite’s method for estimating the degrees of freedom. If significant, this was followed by posthoc testing. For experiments investigating the effect of psilocin dose in WT mice, posthoc Tukey’s Honest Significant Difference or Dunnett’s tests were used. Experiments comparing WT and β-arrestin2 KO mice used posthoc Šídák’s test.

For experiments on cultured primary neurons, the Sholl plot AUC was calculated (AUC function of GraphPad Prism). Values for Sholl plot AUC, NMax, total number of branches and total neurite length were plotted on GraphPad Prism, and groups analyzed statistically using a one- or two-way ANOVA, and where this was significant, posthoc Dunnett’s, Tukey’s or Šídák’s multiple comparison tests as appropriate.

Data are presented as mean ± SEM values with individual values typically indicated by symbols. *p* < 0.05 was considered statistically significant.

## Supplementary Material



## Notes

Additional experimental details, effect of MDL-100,907 on head-twitch response, post-hoc gene expression analysis, additional dendritogenesis measurements (PDF)
