# Supplementary material for: Role of β‑Arrestin2 Signaling in Neuropharmacological Effects of Psychedelic Drugs
Source: ACS Chem Neurosci. 2026 Jul 27;17(15):2971–81. doi: 10.1021/acschemneuro.6c00138 (PMC13449766; doi:10.1021/acschemneuro.6c00138)
Supplement: Supplementary file 1 [file cn6c00138_si_001.pdf]

Supporting Information for Publication

Role of β-arrestin2 signalling in neuropharmacological effects of psychedelic drugs

Aurelija Ippolito, Agnese Tiranti, [1]Shaun Hurley, [1]Gary Gilmour, Grant Churchill and Trevor Sharp

University Department of Pharmacology, Mansfield Road, Oxford, U.K. and [1]Compass Pathways plc, London W1F 0DQ, UK

Correspondence: Aurelija Ippolito (aurelija.ippolito@univ.ox.ac.uk) and Trevor Sharp (trevor.sharp@pharm.ox.ac.uk)

Sex effects throughout experiments:

Experiments were conducted on both male and female mice so the effect of sex on psilocin-induced effects in WT mice were tested. For the head-twitch response, although there was a sex effect, with female mice displaying a greater number of head-twitches than male mice, post-hoc tests revealed no significant increase in the number of head-twitches in female mice compared to male mice at any dose. For plasticity-related gene expression, there was a significant effect of sex for c-Fos only, with males showing higher expression than females but post-hoc tests revealed no significant differences at any dose. Data from the lab has also found a lack of sex effects in DOI-dosed WT mice (unpublished). Given the lack of sex effects seen in WT mice, effects of sex were not analysed in WT vs β-arrestin2 KO experiments.

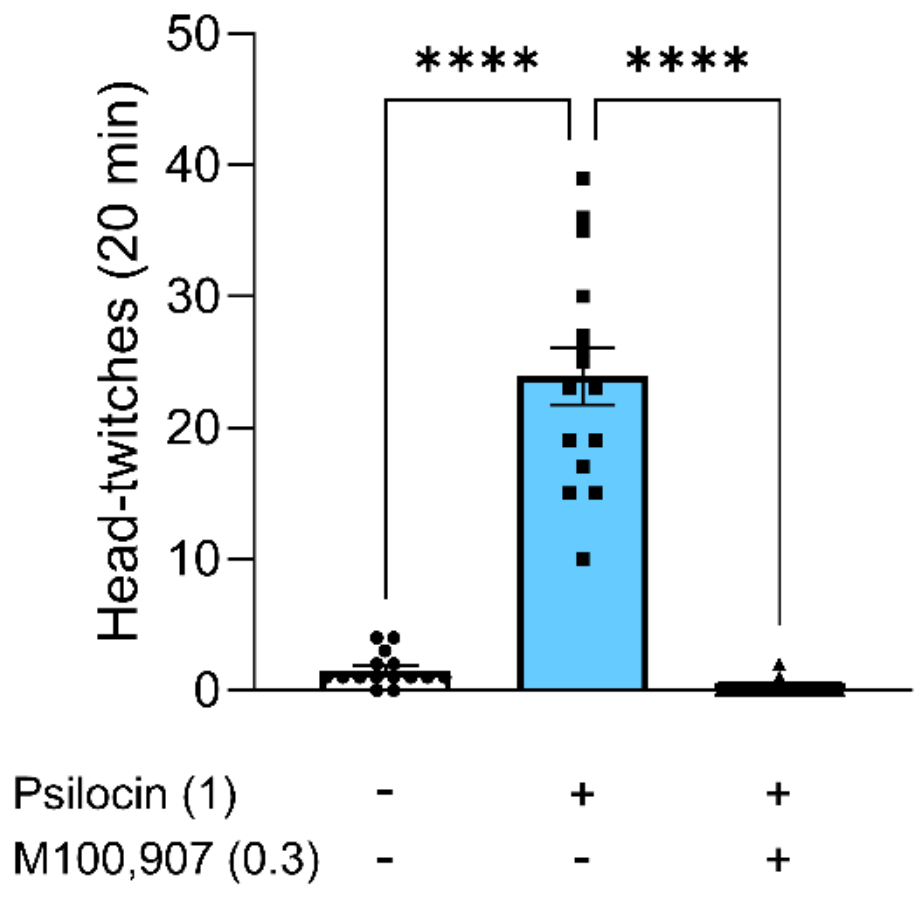

Supplementary Figure 1 | Effect of MDL-100,907 on psilocin-induced head-twitches. Effect of pre-treatment with 0.3 mg/kg MDL-100,907 on the number of head-twitches after vehicle or 1 mg/kg psilocin (n=15-16). Columns represent mean ± SEM values, with individual values indicated by symbols. Data were analysed by a mixed effect model and type III ANOVA and if significant, followed by post-hoc Tukey's Honest Significant Difference or Šídák's tests. ****p<0.0001.

Supplementary Table 1 | Post-hoc analysis of gene expression data for wildtype mice dosed with psilocin. Given the significant effect of drug on expression of *c-Fos*, *Egr-1*, *Egr-2* and *Arc*, post-hoc Dunnett's tests were carried out comparing 1 mg/kg, 2 mg/kg and 5 mg/kg psilocin to vehicle. *p<0.05, **p<0.01, ***p<0.001. Group sizes are as in Fig. 4.

c-Fos			
**Contrast**	**Estimate**	**SE**	**p value**
Vehicle – 1 mg/kg	0.427	0.129	0.00470**
Vehicle – 2 mg/kg	0.291	0.129	0.0743
Vehicle – 5 mg/kg	0.432	0.131	0.00490**

Egr-1			
**Contrast**	**Estimate**	**SE**	**p value**
Vehicle – 1 mg/kg	0.220	0.0805	0.0241*
Vehicle – 2 mg/kg	0.226	0.0805	0.0194*
Vehicle – 5 mg/kg	0.0964	0.0817	0.503

Egr-2			
**Contrast**	**Estimate**	**SE**	**p value**
Vehicle – 1 mg/kg	0.533	0.136	0.0008***
Vehicle – 2 mg/kg	0.368	0.136	0.0255*
Vehicle – 5 mg/kg	0.307	0.138	0.0811

Arc			
**Contrast**	**Estimate**	**SE**	**p value**
Vehicle – 1 mg/kg	0.2575	0.0902	0.0171*
Vehicle – 2 mg/kg	0.2013	0.0902	0.0789
Vehicle – 5 mg/kg	0.0411	0.0915	0.916

Supplementary Table 2 | Post-hoc analysis of the effect of drug on gene expression data in wildtype and β-arrestin2 KO mice dosed with psilocin. Given the significant effect of drug on expression of *c-Fos*, *Egr-1*, *Egr-2* and *Arc*, post-hoc Šídák's tests were carried out comparing WT vs KO mice dosed with 1 mg/kg psilocin. *p<0.05. Group sizes are as in Fig. 4.

c-Fos			
**Genotype**	**Estimate**	**SE**	**p value**
WT	-0.405	0.178	0.0281*
β-arrestin2 KO	-0.172	0.181	0.347

Egr-1			
**Genotype**	**Estimate**	**SE**	**p value**
WT	-0.264	0.126	0.0427*
β-arrestin2 KO	-0.287	0.131	0.0341*

Egr-2			
**Genotype**	**Estimate**	**SE**	**p value**
WT	-0.442	0.167	0.0113*
β-arrestin2 KO	-0.172	0.167	0.309

Arc			
**Genotype**	**Estimate**	**SE**	**p value**
WT	-0.319	0.143	0.0302*
β-arrestin2 KO	-0.265	0.145	0.0733

Supplementary Table 3 | Post-hoc analysis of the effect of drug on gene expression data in wildtype and β-arrestin2 KO mice dosed with DOI. Given the significant effect of drug on expression of *c-Fos*, *Egr-1*, *Egr-2* and *Arc*, post-hoc Šídák's tests were carried out comparing WT vs KO mice dosed with 2 mg/kg DOI. *p<0.05, **p<0.01, ***p<0.001, ****p<0.0001. Group sizes are as in Fig. 5.

c-Fos			
**Genotype**	**Estimate**	**SE**	**p value**
WT	-0.667	0.272	0.0202*
β-arrestin2 KO	-0.790	0.220	0.00110**

Egr-1			
**Genotype**	**Estimate**	**SE**	**p value**
WT	-0.372	0.107	0.00160**
β-arrestin2 KO	-0.500	0.0882	<0.0001****

Egr-2			
**Genotype**	**Estimate**	**SE**	**p value**
WT	-0.665	0.206	0.00310**
β-arrestin2 KO	-0.870	0.167	<0.0001****

Arc			
**Genotype**	**Estimate**	**SE**	**p value**
WT	-0.475	0.160	0.00580**
β-arrestin2 KO	-0.502	0.130	0.0005***

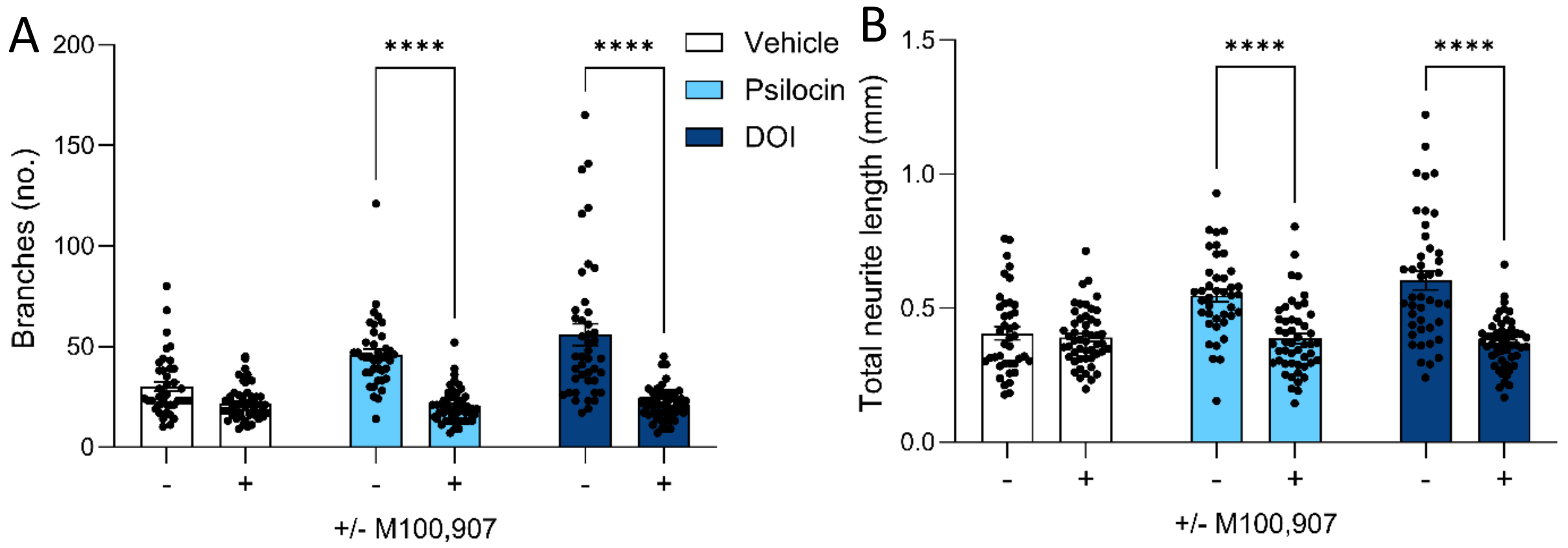

Supplementary Figure 2 | Measurements of dendritic growth of neurons pre-treated with MDL-100,907 before treatment with psychedelic drugs. (A) Total number of branches (B) Total neurite length. n = 39-55 neurons. Columns represent mean ± SEM values, with individual values indicated by symbols. Data were analysed by a two-way ANOVA and if significant, followed by post-hoc Tukey's multiple comparisons tests. ****p<0.0001.

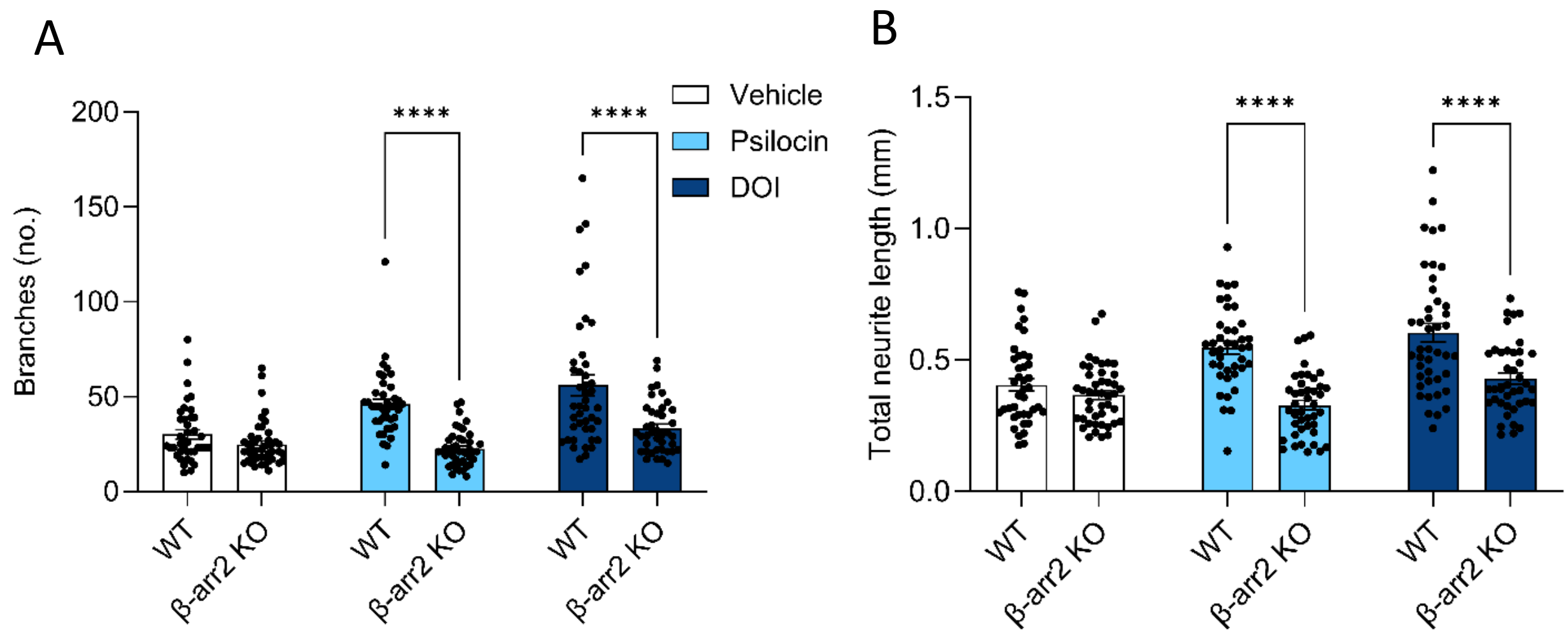

Supplementary Figure 3 | Measurements of dendritic growth of WT and β-arrestin2 KO neurons treated with psychedelic drugs. (A) Total number of branches (B) Total neurite length. n = 39-55 neurons. Columns represent mean ± SEM values, with individual values indicated by symbols. Data were analysed by a two-way ANOVA and if significant, followed by post-hoc Šídák's multiple comparisons tests. ****p<0.0001.

Supplementary Table 4 | Details of the primer sequences used for qPCR analysis.

**Gene**	**Forward primer (5' to 3')**	**Reverse primer (5' to 3')**	**Sequence source**	**Primer efficiency**
c-Fos	AGGGGCAAAGTAGAGCAGCTA	CAATCTCAGTCTGCAACGCA	PrimerBank (ID 31560587b2)	96.04
Egr-1	TTGTGGCCTGAACCCCTTTT	AGATGGGACTGCTGTCGTTG	(Guo et al., 2014)	95.79
Egr-2	TGCTAGCCCTTTCCGTTGA	TCTTTTCCGCTGTCCTCGAT	(Ma et al., 2015)	96.30
Arc	GGAGGGAGGTCTTCTACCGTC	CCCCCACACCTACAGAGACA	PrimerBank (ID 86604725c2)	90.26
BDNF	TTACCTGGATGCCGCAAACAT	TGACCCACTCGCTAATACTGTC	PrimerBank (ID 114326460c3)	95.67
Homer1a	ACCAGTACCCCTTCACAGGA	TGATTGCTGAATTGAATGTGTACCT	NCBI	96.28
Arrb1	CACTGACACGGCTACAGGAG	TGCAGAGTGACTGAGCATGG	(Zhang et al., 2018)	96.28
Arrb2	AGTCGAGCCCTAACTGCAAG	ACGAACACTTTCCGGTCCTTC	(Yang et al., 2019)	100.16
Htr2a	CAGGCAAGTCACAGGATAGC	TTAAGCAGAAAGAAAATCCCACAG	(Couch et al., 2015)	100.00
Gapdh	AGTGCCAGCCTCGTCCCGTAG	GTGCCGTTGAATTTGCCGTGAGTG	(Piazza et al., 2017)	78.53
Actb	CTCTGGCTCCTAGCACCATGAAGA	GTAAAACGCAGCTCAGT	(Stephens et al., 2011)	83.50
